# Antioxidant Defense Expressed as Glutathione Status and Keap1-Nrf2 System Action in Relation to Anthropometric Parameters and Body Composition in Young Women with Polycystic Ovary Syndrome

**DOI:** 10.3390/antiox12030730

**Published:** 2023-03-16

**Authors:** Magdalena Chełchowska, Justyna Jurczewska, Joanna Gajewska, Joanna Mazur, Dorota Szostak-Węgierek, Ewa Rudnicka, Jadwiga Ambroszkiewicz

**Affiliations:** 1 Department of Screening Tests and Metabolic Diagnostics, Institute of Mother and Child, Kasprzaka 17a, 01-211 Warsaw, Poland; 2 Department of Clinical Dietetics, Faculty of Health Sciences, Medical University of Warsaw, E Ciołka Str. 27, 01-445 Warsaw, Poland; 3 Department of Humanization in Medicine and Sexology, Collegium Medicum, University of Zielona Gora, 65-729 Zielona Gora, Poland; 4 Department of Gynecological Endocrinology, Medical University of Warsaw, Karowa 2, 00-315 Warsaw, Poland

**Keywords:** polycystic ovary syndrome, glutathione, glutathione peroxidase, glutathione reductase, obesity, oxidative stress, nuclear factor erythroid 2-related factor 2

## Abstract

Metabolic disorders present in women with polycystic ovary syndrome (PCOS) and the associated risk of obesity may result in increased oxidative stress and reproductive failure. Therefore, we evaluated the concentrations of reduced glutathione (GSH), oxidized glutathione (GSSG), glutathione peroxidase (GPx), and reductase (GR), as well as nuclear factor erythroid 2-related factor 2 (Nrf2) and Kelch-like ECH-associating protein1 (Keap1) in the serum of 56 women with PCOS divided according to the visceral to subcutaneous fat surface ratio (VAT/SAT) and waist-to-hip ratio (WHR) values. Antioxidant parameter levels were measured by competitive inhibition enzyme immunoassay technique. As the VAT/SAT ratio and WHR increased, we observed significantly higher concentrations of GSSG and Keap1 protein and a lower value of the GSSG/GSH ratio (R-index), which is considered an index of cellular redox (*p* < 0.05). Negative correlations were found between the R-index and body weight, BMI, WHR, subcutaneous and visceral fat surface and the VAT/SAT ratio, and total body fat; positive links were found with fat free mass and total body water. Opposite associations were noted between GSSG level and the aforementioned body composition parameters. Oxidative stress characterized by a depleted reduced-to-oxidized glutathione index is associated with anthropometric and body composition parameters in women with PCOS. In particular, abdominal obesity expressed by the VAT/SAT ratio and/or WHR seems to have a negative impact on glutathione status, which may lead to a disruption of many biological cell processes. The observed negative association of Keap1 with R-index suggests that the elevated oxidative changes dependent on the VAT/SAT ratio may lead to Nrf2 activation to promote antioxidant enzyme expression. Although the GSH/GSSG index as well as the VAT/SAT ratio appear to be good indicators of oxidative status, studies on a larger group of patients should continue to confirm these links among women with PCOS.

## 1. Introduction

Polycystic ovary syndrome (PCOS) is one of the most common endocrinopathies and its prevalence—depending on the adopted diagnostic criteria and the population studied—ranges from 4 to 16% of reproductive-age women [1,2,3]. Characteristic features of the syndrome include menstrual disorders, hyperandrogenism, and often obesity and infertility [4,5,6]. In addition to primary and secondary infertility, pregnancy complications are more common in this patient group, including gestational diabetes, hypertension, preeclampsia, and a higher risk of miscarriage or low-birth-weight offspring [7,8,9]. 

It is estimated that up to 60–70% of patients with PCOS are overweight or obese, with particularly increased central distribution of adipose tissue. Central accumulation of adipose tissue is characteristic of both lean and obese women with this disease entity [1]. Visceral adipose tissue is a highly active endocrine organ secreting hormonally active proteins—adipokines, pro-inflammatory cytokines and growth factors involved in the regulation of energy homeostasis, and carbohydrate and lipid metabolism [10,11]. The adipose tissue of women with PCOS shows many abnormalities in the secretion of these compounds, which affect the clinical signs of the disease and exacerbate existing endocrine and metabolic disorders, often leading to reproductive failure [2,3,12].

**Figure 1:**
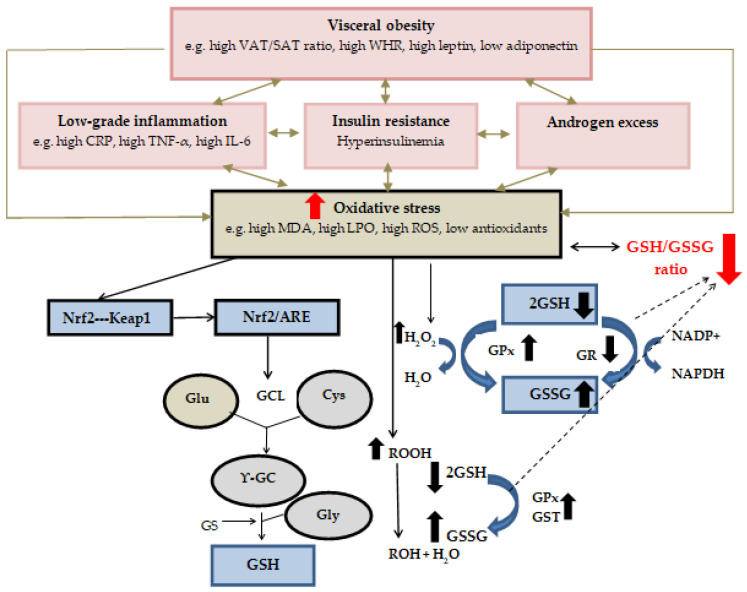
Possible links between oxidative stress and glutathione status and the Keap1-Nrf2 system action in women with polycystic ovary syndrome. Oxidative stress leads to the upregulation of the Keap1-Nrf2 system, resulting in the expression of, e.g., glutamate-cysteine ligase—the rate-limiting enzyme in glutathione synthesis. Oxidative stress dysregulated the glutathione regeneration enzymatic process, resulting in decreased GSH/GSSG ratio levels. Nrf2 = nuclear factor erythroid-derived 2-like protein 2; Keap1 = Kelch-like ECH-associated protein 1; ARE = antioxidant response element; GCL = glutamate-cysteine ligase; GS = glutathione synthetase; Cys = cysteine; Glu = glutamate; Gly = glycine; γGC = γglutamyl-cysteine; GSH = reduced glutathione; GSSG = oxidized glutathione; GPx = glutathione peroxidase; GR = glutathione reductase; GST = glutathione transferase; GSH/GSSG ratio = index cellular redox; CRP = C-reactive protein; TNF-α = tumor necrosis factor α; IL-6 = interleukin 6; VAT = visceral fat surface; SAT = subcutaneous fat surface; WHR = waist-to-hip ratio; MDA = malondialdehyde; LPO = lipid peroxidation; ROS = reactive oxygen species.

Insulin resistance in PCOS resulting in hyperglycemia and higher levels of free fatty acids can lead to increased reactive oxygen species (ROS) production, especially when accompanied by overweight and obesity. Because PCOS is also associated with reduced antioxidant levels, it is considered a state of increased risk for oxidative stress. Research conducted in this area suggests that there may be a strong relationship between impaired adipose tissue metabolism, insulin resistance, hyperandrogenism, inflammation, and oxidative stress in the pathogenesis of PCOS [1,2,3,13,14,15,16,17,18] (Figure 1).

A commonly known measure of oxidative stress is the ratio of reduced glutathione (GSH) to oxidized glutathione (GSSG). The GSH/GSSG system is the main “redox buffer” that protects cellular structures from the damaging effects of free oxygen radicals, and the reactivity of this compound is conditioned by the presence of a thiol group. Glutathione in reduced form in the presence of glutathione peroxidase (GPx) reacts with hydrogen peroxide (H_2_O_2_) and lipid peroxide, oxidizing to disulfide. Regeneration of the active thiol form occurs with the participation of NADPH-dependent glutathione reductase (GR), belonging to flavoproteins. In addition to the regeneration of GSH from GSSG, the second process affecting the increase in glutathione concentration is its neosynthesis. The de novo synthesis of GSH in the cell is limited by the availability of its constituent amino acids, and in particular by the availability of the sulfur amino acid precursor, cysteine [19,20]. Modification of the oxidation state of protein cysteine residues is significantly responsible for the role of GSH in redox-dependent cell signaling. The process of protein glutathionylation is a mechanism that protects sensitive protein thiols from irreversible oxidation, and thus from irreversible loss of their biological activity [18]. One of the transcription factors involved in cell signaling and containing selected cysteine residues is nuclear factor erythroid 2-related factor 2 (Nrf2). Under physiological conditions, Nrf2 exists in the cytoplasm in the form of a complex with Keap1 protein (Kelch-like ECH-associating protein 1). Reactive oxygen and nitrogen species (RNS) formed in excess can oxidize the specific cysteine residues of Keap1, leading to a conformational change of this protein, the release of Nrf2, and its translocation to the cell nucleus. Through the activation of the antioxidant response element (ARE) in the nucleus, it participates in the transcriptional regulation of many antioxidant genes—including, i.e., glutathione S-transferase, NAPH-oxidoreductase, and glutamate-cysteine ligase, the rate-limiting enzyme in glutathione synthesis [19,21,22]. Given the important role of GSH in cellular defense mechanisms, the induction of regulatory enzymes involved in its synthesis plays a key role in protecting cells from excessive oxygen free radical activity [20].

According to the meta-analysis conducted by Murri et al. [1], patients with PCOS had approximately 50% lower glutathione levels compared with healthy women and no changes in glutathione peroxidase activity. There are no systematic data on the relationships between glutathione status and adipose tissue and the risk of metabolic disorders in women with PCOS. There are also limited reports on the Keap1-Nrf2 system action in this disease, and these mainly concern animal models [23,24].

In this study, we aimed to evaluate glutathione status and the function of the Keap1-Nrf2 system in relation to anthropometric parameters and body composition in young women with polycystic ovary syndrome. Therefore, the serum concentrations of GSH, GSSG, GPx and GR, as well as the values of Nrf2 and Keap1 proteins in patients with PCOS, were determined. The interrelationships between the tested antioxidants and the link between antioxidants and body composition parameters were also investigated.

## 2. Materials and Methods

### 2.1. Participants 

The study was conducted in accordance with the ethical standards established by the Declaration of Helsinki after obtaining approval from the Ethical Committee of the Medical University of Warsaw (consent no. KB/170/2019). All participants were acquainted with the objectives and procedures of the study. Patients gave written consent for the analysis of biological samples, anthropometric and body composition measurements, and the use of clinical information collected from their medical records.

The study included 56 Caucasian women with polycystic ovary syndrome, recruited in the Department of Gynecological Endocrinology of the Medical University in Warsaw in the years 2021–2022. The inclusion criteria for the study group were PCOS diagnosed according to the Rotterdam diagnostic criteria (presence of at least two of the following three criteria: oligo-/amenorrhea, clinical and/or biochemical hyperandrogenism, image of polycystic ovary according to ultrasound exam) [25]. Exclusion criteria included: diabetes, chronic hypertension, cardiovascular diseases, thyroid dysfunction, endometriosis, congenital adrenal hyperplasia, Cushing’s syndrome, androgen releasing tumor, use of lipid-lowering or insulin-sensitizing drugs, exacerbated state of chronic and acute somatic disease and/or contagious diseases, mental disorders, genetic defects, pregnancy, and lactation. Due to the method of body composition measurement (BIA—bioelectrical impedance analysis), the additional exclusion criteria were: diagnosed epilepsy, implanted pacemaker or defibrillator, and metal endoprostheses. For further analysis, the patients were divided into two groups according to their visceral to subcutaneous fat surface ratio and the waist-to-hip ratio values.

### 2.2. Anthropometric Measurements 

Body weight and height were measured according to the established standards [26]. Body mass index (BMI) was calculated as the ratio between body weight and height squared (kg/m^2^). Interpretation of these results followed the international classification provided by the World Health Organization (WHO): <18.5kg/m^2^ = underweight; 18.5–24.9 kg/m^2^ = normal weight; 25.0–29.9 kg/m^2^ = overweight; ≥30.0 kg/m^2^ = obese [27].

In addition, waist and hip circumference were measured. According to the WHO recommendations [28], waist circumference was measured at the midpoint between the lower margin of the least palpable rib and the top of the iliac crest, using a stretch-resistant tape. Hip circumference was measured around the widest part of the buttocks. The cut-off point for high values of the waist circumference was >80 cm. Additionally, to measure abdominal obesity, the waist-to-hip ratio (WHR) was calculated by dividing the waist circumference by the hip circumference. Abdominal obesity was defined as WHR ≥ 0.85 [28].

### 2.3. Body Composition Analysis with Bioelectrical Impedance 

Whole body composition of the women was measured using a Maltron BioScan 920-II multi-frequency bioelectrical impedance analyzer according to the manufacturer’s instructions (Maltron International Ltd., Rayleigh, UK). Body composition analysis was performed in the supine position with the limbs separated by 30° from the body. Before starting the examination, the participants were recommended to rest for about three minutes. The electrodes were placed on the top middle part of the right hand and on the top middle part of the right foot. Before placing the electrodes, the placement sites were cleaned using isopropyl alcohol to limit possible errors and ensure adherence.

Quantitative analysis of abdominal adipose tissue (subcutaneous and visceral) was performed in a standing position with the upper limbs separated from the body. The configuration of electrode placement was strictly defined by the device’s manufacturer [29]. Based on the anthropometric measurements and body composition tests, the following parameters were determined: subcutaneous fat surface (SAT in cm^2^), visceral fat surface (VAT in cm^2^), and the ratio of visceral to subcutaneous fat (VAT/SAT ratio). The cut-off for VAT was >120 cm^2^ and that for SAT was >225 cm^2^. At the same time, a VAT/SAT ratio above 0.90 was adopted as a risk factor for metabolic diseases [29]. The obtained results were processed using the Maltron BioScan 920 v. 1.1.135 software. According to the guidelines of the European Society of Parenteral and Enteral Nutrition (ESPEN) for body composition tests, the subjects had to meet the following conditions: entering the test on an empty stomach, emptying the bladder 30 min before the test, lack of physical activity for 12 h before the test, no alcohol and no fluids containing caffeine for 24 h before the test [30].

### 2.4. Biochemical Analysis

For biochemical analysis, 5 mL of venous blood was collected in the morning from all participants after 12 h of overnight fasting during the follicular phase between days two and six of their menstrual cycle. 

Blood samples were prepared in a manner appropriate for the planned biochemical analyses. The serum samples obtained after centrifugation were divided into small portions, some of which were used for testing on the day of collection, and the remaining part was frozen at −80 °C until the rest of the biochemical analyses were performed (stored no longer than three months). 

Serum fasting glucose concentrations were determined by enzymatic reference method with hexokinase using commercial kits on Integra 400 plus a biochemical analyzer (Roche Diagnostics, Basel, Switzerland). 

Serum insulin, testosterone (T), luteinizing hormone (LH), follicle stimulating hormone (FSH), thyroid stimulating hormone (TSH), estradiol (E2), androstenedione (A), 17-hydroxyprogesterone (17-OHP), dehydroepiandrosterone sulfate (DHEA-S), and sex hormone binding globulin (SHBG) levels were measured using one- or two-step chemiluminescent microparticle immunoassay (CMIA; Alinity I analyzer, Abbott Diagnostics GmbH, Wiesbaden, Germany).

To determine insulin resistance, the homeostasis model assessment for insulin resistance (HOMA-IR) was calculated using the following formula: HOMA-IR = [fasting insulin (µU/mL) × fasting glucose (mmol/L)]/22.5.

Serum concentrations of antioxidant defense parameters were measured using immunoenzymatic methods following the manufacturer’s instructions.

GSH and GSSG levels were determined using kits based on a sandwich enzyme-linked immunosorbent assay (ELISA) with two specific and high affinity monoclonal antibodies (Human GSH ELISA Kit Cat. No.: 201-12-5407; Human GSSG ELISA Kit Cat. No.: 201-12-5444, SunRed Bio-technology Company, Shanghai, China), which we have previously described in detail [31]. To assess the cellular redox index, the GSH/GSSG ratio was calculated.

Nuclear factor (erythroid-derived 2)-like 2, also known as NFE2L2 or Nrf2 was measured using human an NFE2L2 ELISA kit (cat. No. EH3417, Fine Biotech Co., Ltd., Wuhan, China) based on the double-antibody sandwich ELISA technique. The pre-coated antibody was an anti-human NFE2L2 monoclonal antibody, while the detection antibody was a biotinylated polyclonal antibody. Samples and biotinylated antibodies were added into ELISA plate wells. Then, avidin–peroxidase conjugates (HRP-streptavidin) were added to the wells. TMB substrate was used for coloration after the enzyme conjugate had already been thoroughly washed out of the wells. TMB (3,3′,5,5′-tetramethylbenzidine) reacts to form a blue product from the peroxidase activity, and finally turns to yellow after adding the stop solution. The color intensity and quantity of target analyte in the sample were positively correlated. The levels of GR, GPx, and Keap1 proteins were determined in an analogous manner using a human glutathione reductase ELISA kit (cat. No. MBS2703164, MyBioSource Inc., San Diego, CA, USA), a human glutathione peroxidase ELISA kit (cat. No. MBS167041, MyBioSource Inc., San Diego, CA, USA), and a human KEAP1 (Kelch-like ECH-associated protein 1) ELISA kit (cat. No. EH4240, Fine Biotech Co., Ltd., Wuhan, China), respectively. The concentrations of NFE2L2, Keap1, GR, and GPx in the samples were calculated by comparing the O.D. of the samples to the standard curve. The intra- and inter-assay CVs were less than 8.0% and 10% for NFE2L2, Keap1, and GPx, whereas they were 10% and 12% for GR, respectively. Assay sensitivity was less than 0.094 ng/mL for NFE2L2, 14.063 pg/mL for Keap1, 0.260 ng/mL for GPx, and 35.000 pg/mL for GR, respectively.

### 2.5. Statistical Analysis

Statistical analysis included 40 parameters in four groups: baseline clinical features (13), anthropometric data and body composition (20), and antioxidant defense parameters (7). The normality of each parameter was checked using the Kolmogorov–Smirnov test. The study group was divided into two according to the visceral to subcutaneous fat surface ratio, with a cut-off point of 0.90, and independently into two groups according to the waist-to-hip ratio values, with a cut-off point of 0.85. For each of the identified subgroups, the mean values of the respective parameters were presented with the standard deviation (SD) if the distribution did not deviate from normal, or the median along with the interquartile range (1-3IQR) if the hypothesis of normality of distribution was rejected. In the first case, the groups were compared using the parametric Student’s *t*-test and in the second case using the nonparametric Mann–Whitney test. The level of correlation between GSH, GSSG, as well as the R-index and anthropometric parameters was also calculated using Spearman’s rho. The relationship between Keap1 and the three aforementioned antioxidant defense parameters are presented graphically as a scatter plot.

All reported p-values were two-tailed, and values = <0.05 were considered significant. IBM SPSS v.28 statistical software was used (IBM SPSS Statistics for Windows, Version 28.0. Armonk, NY, USA: IBM Corp). 

## 3. Results

Table 1 shows a comparison of the clinical characteristics of the 56 participants with PCOS stratified twice: once based on the VAT/SAT ratio (≤0.90; >0.90) values and a second time based on the WHR values (<0.85; ≥0.85). Patients in each group were of similar age (median 25 years). We observed significantly higher levels of fasting insulin and HOMA-IR values in the serum of women with increased VAT/SAT index (VAT/SAT > 0.90) and WHR ratio (WHR ≥ 0.85) when compared with women with normal values of these parameters (*p* < 0.01). Fasting glucose levels were also higher in these groups, but the difference was not statistically significant. 

We found that patients with higher VAT/SAT and WHR ratios had significantly lower levels of SHBG than patients with VAT/SAT ≤ 0.90 (*p* < 0.001) and WHR < 0.85 (*p* < 0.01). Additionally, lower VAT/SAT values were associated with lower levels of 17-OHP (*p* < 0.01). We did not observe significant differences in LH, FSH, T, E2, A, TSH, and DHEAS concentrations between the studied groups. 

As expected, anthropometric and body composition indices differed significantly in both groups divided by VAT/SAT and WHR values. The PCOS group with increased VAT/SAT ratios had statistically higher subcutaneous and visceral adipose tissue content, BMI, total body fat, and muscle mass compared with the PCOS group with normal VAT/SAT ratio (*p* < 0.001). Additionally, this group of women was characterized by a significantly higher WHR compared with women with VAT/SAT ≤ 0.90 (*p* < 0.001). Similar relationships were observed in the group of women with WHR ≥ 0.85. The subjects’ detailed anthropometric data and body composition measures are shown in Table 2. 

Serum antioxidant parameters in women with PCOS from each subgroup are summarized in Table 3 and Table 4. We found that GSSG and Keap1 protein concentrations were statistically higher, while the R-index value was significantly lower in the serum of women with increased VAT/SAT compared with the group with normal values of this ratio (*p* < 0.001; Table 3). 

Similar differences were shown for GSSG and R-index between the WHR ≥ 0.85 and WHR < 0.85 groups (*p* < 0.05 and *p* < 0.01, respectively; Table 4). Other antioxidant defense parameters were not significantly different between all the studied groups. As the VAT/SAT and WHR ratio increased, we observed lower concentrations of reduced glutathione; however, these differences were not statistically significant (although in the case of the WHR ratio, they were at the limit of significance *p* = 0.053; Table 3 and Table 4).

The correlations of GSH, GSSG, and the R-index with anthropometric parameters and body composition in women with PCOS are shown in detail in Table 5 (whole group). Serum GSH concentrations were negatively correlated with hip circumference, WHR, and VAT/SAT ratio values. Serum GSSG levels were positively associated with weight, BMI, WHR, subcutaneous and visceral adipose tissue content, the VAT/SAT ratio, total body fat, and cell mass, and inversely correlated with fat-free mass and total body water. In contrast, we found negative correlations of R-index values with weight, BMI, WHR, subcutaneous and visceral adipose tissue content, the VAT/SAT ratio, and total body fat mass, whereas we found a positive correlation of R-index values with fat-free mass and total body water. We also observed positive relations between Keap1 and VAT/SAT ratio values (r = 0.263; *p* = 0.05).

Increased concentrations of insulin and HOMA-IR levels observed in women with PCOS were positively correlated with oxidized glutathione concentrations (r = 0.418, *p* = 0.001; r = 0.405, *p* = 0.002, respectively) and negatively correlated with R-index values (r = −0.304, *p* = 0.003; r = −0.380, *p* = 0.004, respectively). In addition, associations between Nrf2 concentrations and insulin (r = 0.256, *p* = 0.057) and HOMA-IR levels (r = 0.260, *p* = 0.053) were on the border of significance.

**Figure 2:**
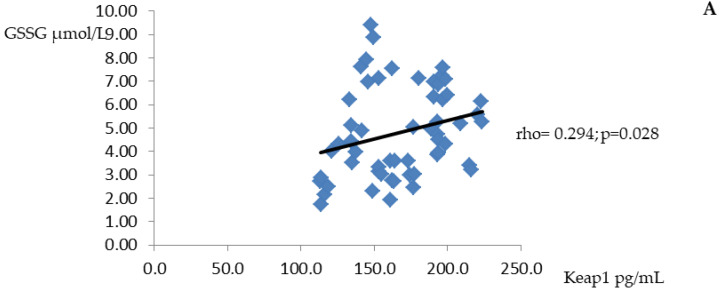

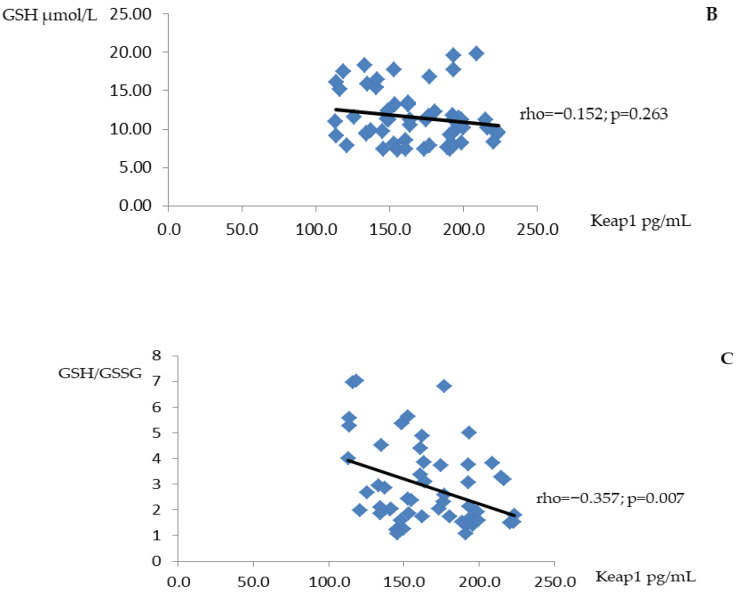
Scatter plot for the relationships between Keap1 and GSSG (**A**), GSH (**B**), as well as R-index (**C**) in women with PCOS.

Figure 2A–C shows the relationships between glutathione status parameters and Keap1 protein in women with PCOS. There was a statistically significant positive correlation between levels of GSSG and Keap1 concentrations (A), no correlations between GSH and Keap1 levels (B), and a significant negative correlation between Keap1 levels and R-index values (C). Additionally, increased values of reduced glutathione to oxidized glutathione ratio were significantly associated with increased levels of glutathione reductase in the serum of patients with PCOS (r = 0.384, *p* = 0.009). Other relationships between the selected antioxidant defense parameters were not confirmed.

## 4. Discussion

Compared with healthy counterparts, body fat distribution is different in women with PCOS due to the predominant accumulation of visceral fat and abdominal obesity [3,12,32]. Accompanying PCOS, abnormal adipose tissue metabolism and associated chronic low-grade inflammation are a significant source of reactive oxygen species [14,33]. Excess free radicals with reduced antioxidant activity—including glutathione, an important small-molecule antioxidant observed in patients with PCOS—may be responsible for exacerbating oxidative stress [18]. 

Studies determining glutathione status in patients with PCOS mainly concern its reduced form or the activity of glutathione peroxidase [1,17]. In the PCOS group, GSH concentrations were lower by half compared with healthy women [16,34,35]. Data on GPx activity are inconclusive and show both lower, higher, and unchanged activity of this enzyme in patients with polycystic ovary syndrome [5,15,17]. The most frequently analyzed relations were glutathione and GPx, with indicators of insulin resistance, hyperandrogenism, and infertility [3,4,18,36]. Associations between parameters of glutathione status and body composition have not been systematically studied and require attention. This is particularly important given that visceral obesity occurs in both overweight and normal-weight women [32]. 

In the present study, we showed differences in the levels of the tested oxidative stress markers both when dividing the groups due to WHR, determining abdominal obesity, and VAT/SAT, determining an additional increased risk of metabolic syndrome. We demonstrated that reduced glutathione was slightly higher in the group of PCOS women with low WHR and VAT/SAT ratios. This finding is consistent with the results obtained by Uckan et al. [35], who found significant differences in the level of this association between nonobese and obese patients with PCOS. Similarly to others, we revealed a close association of glutathione with WHR and the VAT/SAT ratio, while we did not confirm an association of GSH with insulin parameters [16,18,35]. As a result of GPx catalyzing the neutralization of toxic H_2_O_2_, reduced glutathione is oxidized to disulfide (oxidized glutathione), which—with the participation of glutathione reductase and NADPH coenzyme—is reduced back to thiol, which is an important redox cycle in the cell [36]. The effect of obesity on GPx activity has not been clearly confirmed [3,35]. Data determining oxidized glutathione levels in patients with PCOS are also unknown. In our study, GSSG levels were significantly higher in patients with elevated WHR and VAT/SAT with unchanged GPx and slightly lower GR levels. Due to the high lipolytic activity of visceral tissue and the release of large amounts of fatty acids and pro-inflammatory cytokines, peroxidative damage and the accumulation of GSSG may be enhanced in patients with PCOS [37]. Oxidized glutathione is a potentially toxic metabolite for cells. Elevated oxidized glutathione concentrations lead to a disruption of the GSH/GSSG ratio, which is crucial for many biological cell processes, such as the regulation of gene transcription or enzyme and receptor activity [36]. In the present study, high GSSG concentrations were accompanied by low GSH/GSSG ratio levels, which may suggest a shift in the balance toward oxidative processes. The close positive associations of GSSG and negative correlations of the GSH/GSSG ratio with most anthropometric data (e.g., WHR, BMI, WC, and HC) and body composition parameters (e.g., VAT/SAT ratio and fat mass in kg and %) may suggest the severity of oxidative stress in PCOS patients with abdominal and visceral obesity. The low R-index value may indicate an impaired process of reducing excessive GSSG to the active form of GSH with the involvement of glutathione reductase. 

In our study, GR levels were the lowest in patients with the highest VAT/SAT ratio, while GR correlated positively with GSSG concentration and negatively with the R-index value. This confirms the important role of this enzyme in the glutathione redox cycle. 

As we noted in the introduction, in addition to the regeneration of GSH from GSSG via GR, the concentration of this compound in cells is dependent on de novo synthesis. The neosynthesis of GSH in the cells is limited by the availability of cysteine, whose oxidative modifications are largely responsible for glutathione’s role in redox-dependent cell signaling. Containing cysteine residues, Nrf2 participates in the transcriptional regulation of many antioxidant genes, including enzymes that regulate the rate of glutathione synthesis. Activation of Nrf2 by pro-oxidant factors or specific Nrf2 activators (e.g., resveratrol, quercetin, and astaxanthin) is associated primarily with conformational changes in the Keap1 inhibitory protein [21,38,39,40]. In a rat model of PCOS, Li et al. [23] confirmed that granulosa cells (GCs) under oxidative stress show high levels of Nrf2 and heme oxygenase-1 (HO-1). Additionally, Wang et al. [24] documented that humanin, a mitochondrial-derived peptide, regulates oxidative stress in the ovaries of patients with polycystic ovary syndrome via the Keap1-Nrf2 pathway. Similarly, Gharaei et al. [21] showed that astaxanthin supplementation in women with PCOS undergoing assisted reproductive techniques positively affected antioxidant status in the blood and Keap1-Nrf2 pathway activation in GCs. 

The links between oxidative stress, glutathione, and the Keap1-Nrf2 system have not yet been described. We observed slightly increased Nrf2 levels in patients with abnormal VAT/SAT and WHR ratios. In addition, in the group with VAT/SAT > 0.90, we found significantly higher Keap1 protein levels. Moreover, the negative association of Keap1 with the R-index may suggest that the elevated oxidative changes observed in this group may lead to Keap1 dissociations from Nrf2 and the activation of this factor to promote antioxidant enzyme expression. The unchanged GPx levels observed in all the groups with PCOS and only slightly reduced GR in the groups with increased risk of metabolic disorders may be the result of a compensatory antioxidant response associated with the activation of the Keap1-Nrf2 system. It is currently known that, in addition to Keap1, there are other factors that can regulate Nrf2 gene transcription. These include phosphorylation of Nrf2 by protein kinases or acetylation of Nrf2 [41,42]. For this reason, our research in this area should be considered preliminary and should be continued to confirm possible links between Nrf2 action and antioxidant response in women with PCOS.

### Strengths and Limitations 

A strength of the presented research was taking comprehensive measurements of glutathione status markers in the blood of women with PCOS in relation to body composition, with particular emphasis on visceral adipose tissue storage. Moreover, determining the ratio of GSH to GSSG allowed us to estimate the severity of oxidative stress in these patients. Assessment of Nrf2 and Keap1 proteins in the serum of patients with PCOS also seems to be important, although, on the other hand, it may be a certain limitation. Serum concentrations of this factor may not reflect the true cellular antioxidant response as Nrf2 functions mainly in the cell nucleus, and released forms do not always represent its free or active status. Another limitation of the study is that we did not measure serum levels of sulfur amino acids, which are important for the synthesis of this compound. However, we are planning to assess the amino acid profile—such as cysteine, cysteamine, cysteinyl-glycine, and homocysteine—in a future study of patients with PCOS. Finally, the lack of lipid profile and inflammatory markers may be a further limitation of our study. However, it is well known that patients with PCOS often have abnormal lipid levels, and chronic low-grade systemic inflammation is an important factor in this disease [6,14,33,43,44].

## 5. Conclusions

In conclusion, oxidative stress characterized by a depleted reduced-to-oxidized glutathione index is associated with anthropometric and body composition parameters in women with PCOS. In particular, abdominal obesity expressed by the VAT/SAT ratio and/or WHR seems to have a negative impact on glutathione status, which may lead to a disruption of many biological cell processes. The observed negative association of Keap1 with the R-index suggests that the elevated oxidative changes dependent on the VAT/SAT ratio may lead to Nrf2 activation to promote antioxidant enzyme expression. Although the GSH/GSSG index, as well as the VAT/SAT ratio, appear to be good indicators of oxidative status, studies on a larger group of patients should continue to confirm these links among women with PCOS.

## Figures and Tables

**Table 1:** Baseline clinical features in women with PCOS divided according to the visceral to subcutaneous fat surface ratio and the waist-to-hip ratio.

Parameters	VAT/SAT ≤ 0.90 *n* = 29	VAT/SAT > 0.90 *n* = 27	*p*-Value	WHR < 0.85 *n* = 35	WHR ≥ 0.85 *n* = 21	*p*-Value
^a^ Age (years)	25.66 ± 4.55	26.30 ± 3.61	0.323	25.60 ± 3.85	26.00 ± 4.51	0.541
^a^ Fasting ^b^ Glucose (mmol/L)	4.98 ± 0.31	5.21 ± 0.43	0.062	5.03 ± 0.33	5.19 ± 0.47	0.323
^b^ Fasting Insulin (μmU/mL)	5.00 3.87–6.25	9.60 7.30–13.80	0.000	5.80 4.20–7.40	8.10 5.44–13.05	0.006
^b^ HOMA-IR Index	1.13 0.83–1.36	2.34 1.62–3.16	0.000	1.29 0.93–1.68	1.87 1.25–2.92	0.008
^b^ LH (mIU/mL)	5.81 4.37–68.00	6.58 3.30–9.26	0.922	5.73 3.89–8.20	8.01 3.25–10.12	0.243
^b^ FSH (mIU/mL)	4.88 4.50–6.47	5.27 4.75–6.28	0.491	4.88 4.48–6.61	5.27 4.73–6.24	0.417
^a^ T (ng/mL)	0.56 ± 0.13	0.53 ± 0.19	0.394	0.52 ± 0.16	0.59 ± 0.15	0.115
^b^ E2 (pg/mL)	29.00 23.50–44.50	34.00 25.00–46.97	0.486	36.99 24.00–42.00	34.62 21.90–49.00	0.576
^b^ DHEAS (μmol/L)	9.11 6.76–12.26	8.51 6.20–15.08	0.812	8.96 6.72–11.81	8.93 6.25–15.56	0.537
^b^ A (ng/mL)	3.39 2.98–3.88	2.59 2.19–3.63	0.054	3.24 2.27–3.68	3.01 2.52–3.91	0.793
^b^ 17-OHP (ng/mL)	1.53 1.24–2.07	1.20 0.83–1.49	0.006	1.47 1.01–1.77	1.23 0.84–1.55	0.170
^b^ TSH (μIU/mL)	1.65 1.22–2.26	1.70 1.07–2.60	0.980	1.70 1.24–2.23	1.63 0.98–2.74	0.819
^b^ SHBG (nmol/L)	62.00 46.50–71.00	28.00 20.00–39.00	0.000	52.00 39.00–67.00	30.00 20.30–46.90	0.002

^a^ Data were analyzed using Student’s *t*-test and presented as mean and standard deviation (SD). ^b^ Data were analyzed using the Mann–Whitney U test and presented as medians and interquartile ranges (1-3IQR). VAT/SAT = ratio of visceral to subcutaneous fat; WHR = waist-to-hip ratio; HOMA-IR = homeostatic model assessment for insulin resistance; LH = luteinizing hormone; FSH = follicle-stimulating hormone; T = testosterone; E2 = estradiol; DHEA-S = dehydroepiandrosterone sulfate; A = androstenedione; 17-OHP = 17-hydroxyprogesterone; TSH = thyrotropin; SHBG = sex hormone binding globulin.

**Table 2:** Characteristics of anthropometric data and body composition in women with PCOS divided according to the visceral to subcutaneous fat surface ratio and the waist-to-hip ratio values.

Parameters	VAT/SAT ≤ 0.90 *n* = 29	VAT/SAT > 0.90 *n* = 27	*p*-Value	WHR < 0.85 *n* = 35	WHR ≥ 0.85 *n* = 21	*p*-Value
^b^ Body Weight (kg)	58.90 54.50–68.00	93.00 81.00–111.00	0.000	59.00 56.00–70.00	91.00 77.5–105.5	0.000
^a^ Height (cm)	166.45 ± 4.35	166.96 ± 5.92	0.987	166.91 ± 5.03	166.33 ± 5.38	0.546
^a^ BMI kg/m^2^	21.73 ± 2.83	33.18 ± 6.05	0.000	23.78 ± 5.91	33.06 ± 5.86	0.000
^b^ WC (cm)	72.00 67.50–78.50	103.00 95.00–113.00	0.000	74.00 68.00–86.00	103.00 95.50–115.00	0.000
^b^ HC (cm)	93.00 88.00–98.50	114 109.00–124.00	0.000	94.00 90.00–104.00	114.00 104.00–123.00	0.000
WHR	0.78 0.76–0.83	0.87 0.84–0.91	0.000	0.80 0.76–0.82	0.88 0.87–0.95	0.000
^b^ VAT (cm^2^)	51.00 31.00–63.00	291.00 184.00–350.00	0.000	51.00 33.00–115.00	301.00 100.00–350.00	0.000
^b^ SAT (cm^2^)	78.00 74.75–198.75	200.00 59.50–110.00	0.000	100.00 68.00–127.00	219.00 150.50–284.50	0.000
^b^ VAT/SAT	0.53 0.47–0.62	1.50 1.19–1.89	0.000	0.57 0.48–0.93	1.59 1.26–1.96	0.000
^b^ FM (kg)	15.03 12.19–21.11	41.97 32.34–49.38	0.000	15.86 12.33–23.60	40.21 28.84–50.54	0.000
^b^ FM (%)	26.28 26.09–43.84	44.09 39.29–46.95	0.000	27.10 22.12–33.71	42.81 37.77–48.00	0.000
^a^ FFM (kg)	44.25 ± 3.11	52.16 ± 5.97	0.000	45.91 ± 5.49	51.65 ± 5.55	0.000
^a^ FFM (%)	74.01 ± 5.98	57.38 ± 6.83	0.000	71.16 ± 8.97	57.38 ± 6.53	0.000
^a^ MM (kg)	19.19 ± 1.56	23.08 ± 2.69	0.000	19.98 ± 2.63	22.74 ± 2.66	0.001
^a^ BCM (kg)	22.21 ± 2.11	28.49 ± 3.88	0.000	23.56 ± 3.88	28.04 ± 3.82	0.000
^a^ ECM (kg)	21.94 ± 1.61	23.68 ± 2.22	0.002	22.28 ± 1.91	23.62 ± 2.19	0.013
^a^ TBW (%)	50.38 ± 3.26	42.37 ± 3.41	0.000	48.93 ± 4.45	42.45 ± 3.62	0.000
^a^ ECW (%)	48.73 ± 4.61	45.45 ± 0.88	0.000	47.69 ± 3.75	46.25 ± 3.63	0.000
^a^ ICW (%)	51.26 ± 4.61	54.58 ± 0.95	0.000	52.33 ± 3.78	53.74 ± 3.63	0.000
^a^ ECW/ICW	0.97 ± 0.26	0.83 ± 0.03	0.000	0.93 ± 0.21	0.97 ± 0.17	0.000

^a^ Data were analyzed using Student’s *t*-test and presented as mean and standard deviation (SD). ^b^ Data were analyzed using the Mann–Whitney U test and presented as medians and interquartile ranges (1-3IQR). VAT/SAT = ratio of visceral to subcutaneous fat; WHR = waist-to-hip ratio; BMI = body mass index; WC = waist circumference; HC = hip circumference; FM = fat mass; FFM = fat-free mass; MM = muscle mass; BCM = body cell mass; ECM = extracellular matrix; TBW = total body water; ECW = extracellular water; ICW = intercellular water.

**Table 3:** Antioxidant defense parameters in women with PCOS divided according to the visceral to subcutaneous fat surface ratio values.

Parameters	VAT/SAT ≤ 0.90 *n* = 29	VAT/SAT > 0.90 *n* = 27	*p*-Value
^a^ GSH (µmol/L)	12.25 ± 3.64	10.74 ± 3.09	0.140
^a^ GSSG (µmol/L)	3.68 ± 1.31	6.04 ± 1.75	0.000
^b^ R (GSH/GSSG)	3.29 2.05–5.13	1.68 1.48–2.38	0.000
^a^ GPx (ng/mL)	17.66 ± 4.99	18.38 ± 4.42	0.512
^b^ GR (pg/mL)	261.00 199.85–331.67	245.56 185.80–301.23	0.181
^a^ Nrf2 (ng/mL)	1.42 ± 0.24	1.56 ± 0.33	0.147
^a^ Keap1 (pg/mL)	158.78 ± 32.26	176.94 ± 28.13	0.042

^a^ Data were analyzed using Student’s *t*-test and presented as mean and standard deviation (SD). ^b^ Data were analyzed using the Mann–Whitney U test and presented as medians and interquartile ranges (1-3IQR). VAT/SAT = ratio of visceral to subcutaneous fat; GSH = reduced glutathione; GSSG = oxidized glutathione; GPx = glutathione peroxidase; GR = glutathione reductase; R (GSH/GSSG ratio) = index of cellular redox; Nrf2 = nuclear factor erythroid-derived 2-like protein 2; Keap1 = Kelch-like ECH-associated protein 1.

**Table 4:** Antioxidant defense parameters in women with PCOS divided according to the waist-to-hip ratio values.

Parameters	WHR < 0.85 *n* = 35	WHR ≥ 0.85 *n* = 21	*p*-Value
^a^ GSH (µmol/L)	12.30 ± 3.77	10.23 ± 2.37	0.053
^a^ GSSG (µmol/L)	4.02 2.99–5.26	6.42 3.61–7.42	0.016
^b^ R (GSH/GSSG)	2.95 1.86–4.40	1.81 1.46–2.41	0.010
^a^ GPx (ng/mL)	18.89 13.44–22.18	15.83 14.09–20.74	0.537
^b^ GR (pg/mL)	268.61 ± 87.68	248.82 ± 69.74	0.412
^a^ Nrf2 (ng/mL)	1.44 ± 0.21	1.52 ± 0.33	0.393
^a^ Keap1 (pg/mL)	168.38 ± 32.78	166.12 ± 29.76	0.800

^a^ Data were analyzed using Student’s *t*-test and presented as mean and standard deviation (SD). ^b^ Data were analyzed using the Mann–Whitney U test and presented as medians and interquartile ranges (1-3IQR). BMI = body mass index; WHR = waist-to-hip ratio; GSH = reduced glutathione; GSSG = oxidized glutathione; GPx = glutathione peroxidase; GR = glutathione reductase; R (GSH/GSSG ratio) = index of cellular redox; Nrf2 = nuclear factor erythroid-derived 2-like protein 2; Keap1 = Kelch-like ECH-associated protein 1.

**Table 5:** Spearman’s rank correlation coefficients between GSH, GSSG, as well as R-index and anthropometric parameters in women with PCOS.

Parameters	GSH		GSSG		R (GSH/GSSG)	
	rho	* p− * Value	rho	* p− * Value	rho	* p− * Value
Weight (kg)	−0.135	0.357	0.323	0.007	−0.305	0.002
Height (cm)	0.077	0.573	−0.089	0.516	0.066	0.629
BMI kg/m^2^	−0.179	0.188	0.371	0.005	−0.331	0.013
WC (cm)	−0.220	0.103	0.350	0.008	−0.332	0.013
HC (cm)	−0.109	0.012	0.335	0.012	−0.274	0.041
WHR	−0.333	0.012	0.298	0.026	−0.357	0.007
VAT (cm^2^)	−0.239	0.076	0.416	0.001	−0.416	0.001
SAT (cm^2^)	−0.207	0.127	0.348	0.009	−0.329	0.013
VAT/SAT	−0.277	0.039	0.471	0.000	−0.504	0.000
FM (kg)	−0.164	0.227	0.396	0.003	−0.351	0.008
FM (%)	−0.172	0.205	0.448	0.001	−0.399	0.002
FFM (kg)	−0.096	0.481	0.238	0.077	0.192	0.156
FFM (%)	0.170	0.209	−0.443	0.001	0.393	0.003
MM (kg)	−0.085	0.532	0.255	0.057	−0.213	0.116
BCM (kg)	−0.092	0.499	0.308	0.021	−0.251	0.062
ECM (kg)	−0.017	0.903	0.081	0.555	−0.050	0.714
TBW (%)	0.176	0.196	−0.451	0.000	0.408	0.022
ECW (%)	0.052	0.704	−0.383	0.004	0.307	0.021
ICW (%)	−0.051	0.711	0.383	0.004	−0.306	0.022
ECW/ICW	0.051	0.711	−0.380	0.004	0.304	0.023

GSH = reduced glutathione; GSSG = oxidized glutathione; R (GSH/GSSG ratio) = index of cellular redox; BMI = body mass index; WC = waist circumference; HC = hip circumference; WHR = waist-to-hip ratio; VAT = visceral fat surface; SAT = subcutaneous fat surface; VAT/SAT = ratio of visceral to subcutaneous fat; FM = fat mass; FFM = fat-free mass; MM = muscle mass; BCM = body cell mass; ECM = extracellular matrix; TBW = total body water; ECW = extracellular water; ICW = intercellular water.

## Data Availability

The data analyzed during the current study are available from the corresponding author on reasonable request.
